# Editorial: Pathophysiology and management of amenorrhea and estradiol deficiency in girls and young women

**DOI:** 10.3389/fendo.2024.1397210

**Published:** 2024-04-22

**Authors:** Lawrence M. Nelson, Joshua Johnson

**Affiliations:** ^1^ Digital Women's Health Initiative, Mary Elizabeth Conover Foundation, Tysons, VA, United States; ^2^ Department of Obstetrics and Gynecology, University of Colorado-Anschutz Medical Campus, Denver, CO, United States

**Keywords:** women’s hormonal health, 17-beta-estradiol, 17-beta-estradiol deficiency, amenorrhea, women’s health advocacy, women’s health policy, primary ovarian insufficiency, physiologic hormone replacement therapy (P-HRT)

## Developmental endocrinology and women’s health

Developmental endocrinology is a fascinating and mature field that investigates the role of hormones in the growth and development of living organisms. Hormones influence various life processes, from the earliest stages of life to old age. As a study of bodily communication at the cellular and humoral level, it integrates all aspects of health. As such, women’s healthcare and research find a comfortable home here. As a multidisciplinary field, developmental endocrinology draws on knowledge and uses tools from several other areas, including developmental biology, genetics, physiology, and neuroscience. All these approaches unlock new insights into interactions involving growth and development, reproduction, metabolism, and behavior, all as influenced by hormonal action. Developmental endocrinology will continue to shed light on how hormones impact health and disease across the lifespan, and its enormous translational value makes it a crucial area of research that supports the health of women. Despite advances in the field, it is important to acknowledge that there has been a shortfall in investment in women’s health overall, including underinvestment in research in the specific context of women’s reproduction.

## A crisis in women’s health care and research

The historical underinvestment in women’s healthcare and research is attracting increasing attention and can be recognized as a core disparity in need of redress. The World Economic Forum has declared that women are second-class citizens when it comes to health. According to their analysis, closing the gap in care could be worth $1 trillion annually by 2040 (1, 2), in part because women’s health and women’s economic development are inextricably linked (3). In one specific example of a critical health disparity, young women experiencing myocardial infarction have an 84.3% higher mortality rate than young men (4). Such an enormously elevated risk of death points toward the critical need to better understand the sex differences in the biology of disease and improve care delivery for women. Consider that the NIH allocates only 11% of its budget to women’s health-specific research in the US (5). Such poor funding for women’s health research has resulted in health gaps like that seen for myocardial infarction that can only be addressed by additional funding and new business models to support gender-appropriate care. Although data about the financing of women’s health research could be more comprehensive and precise, public funding is the primary support mechanism. Creative ways to bolster funding are being considered across public and commercial research channels (6–10).

One report within this Research Topic, *A Call for a US National Institute of Women’s Health and Human Development* (Nelson), calls for a social enterprise approach to women’s health to synergize with government funding. Private equity and venture capital investments in women’s health are proliferating as opportunities in women’s health become more evident and more technology startups by women set out to disrupt the healthcare market. Within the feminine tech (“FemTech”) space, there is a concentration of activity concerning hormone replacement, consumer menstrual products, gynecological devices, fertility solutions, and maternal health patient support (7).

**Figure 1:**
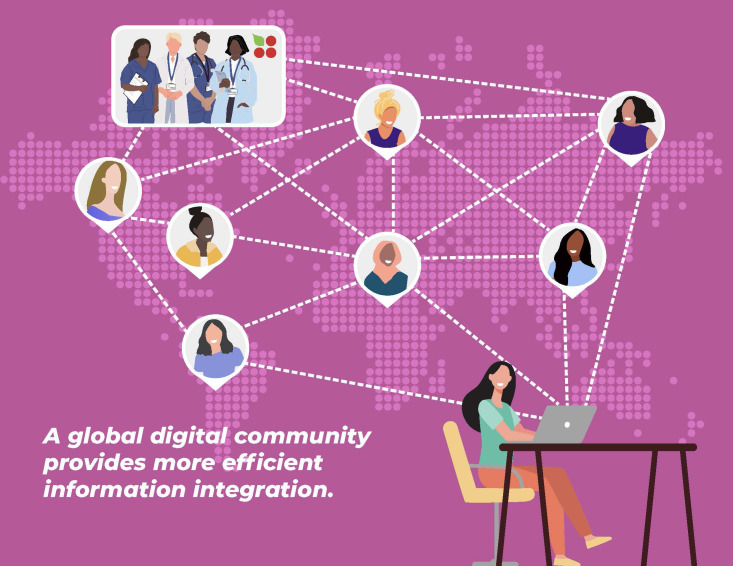
A global digital community provides more efficient information integration (6).

Another report herein, *My 28 Days - a global digital women’s health initiative for evaluation and management of secondary amenorrhea: case report and literature review* (Nelson et al.), suggests that digital health is another potential avenue for innovation, with the potential to make health more equitable (6) (**Figure 1**). Feminine tech companies received only 3% of the total digital health funding, indicating the need for more investment in this space (7). Given the significant unmet need and resulting opportunity, those who continue to forgo investing in women’s health may find themselves left behind by the players that tap into this high-potential market. Having summarized the information in the above reports about the need for more and more thoughtful investment in women’s health research, we turn to the Research Topic’s entries that mainly focus on the endocrinology of ovarian insufficiency.

In *Chronic and Cumulative Adverse Life Events in Women with Primary Ovarian Insufficiency: An Exploratory Qualitative Study* (Sun et al.), the authors’ findings are consistent with the hypothesis that adverse life events play a role in the development of POI (5). Linking adverse life events with the pathophysiological outcome of accelerated ovarian demise is a direct example of how “women’s health and women’s economic [status] are inextricably linked,” considering that socioeconomic status can strongly correspond to the incidence of adverse life events (11). The authors indicate a need for more research in this area.

## 17-beta-estradiol deficiency

**Figure 2:**
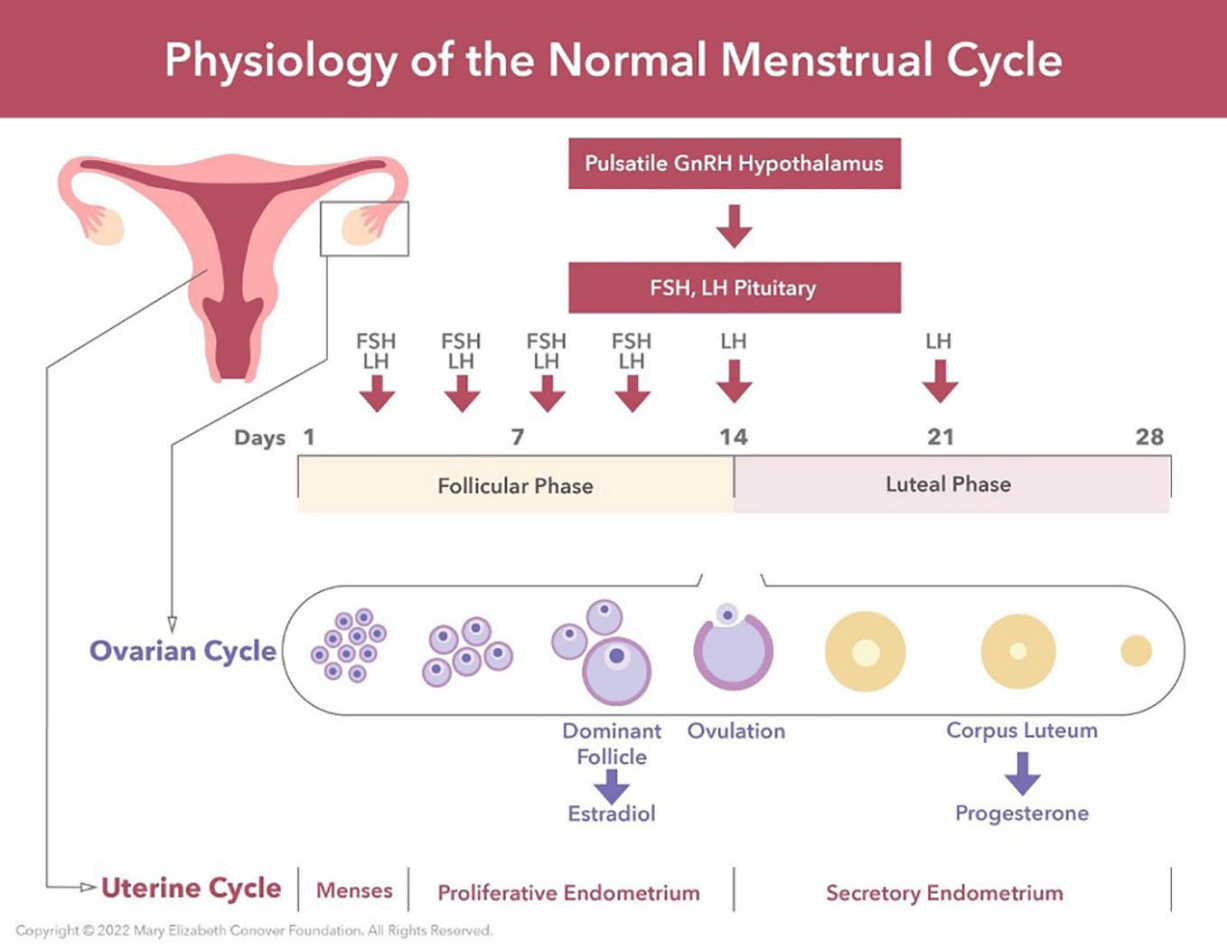
Menarche and the maintenance of normal regular menses require normal function of the hypothalamic-pituitary-ovarian axis. GnRH neurons located in the hypothalamus produce regular pulses of GnRH at set intervals determined by estradiol and progesterone levels. These GnRH pulses are required for the synthesis, storage, and release of FSH and LH by the anterior pituitary. Estradiol provides negative feedback to the central axis during follicular growth. This then switches to positive feedback under the influence of the preovulatory estradiol surge. This positive feedback induces the LH surge, which in turn induces ovulation. My 28 Days - a global digital women’s health initiative for evaluation and management of secondary amenorrhea: case report and literature review (Nelson et al.).

A unifying theme of several entries in this Research Topic is the hormone 17-Beta-Estradiol, the most potent naturally circulating and clinically significant estrogen. As noted in *The Truth about 17-Beta Estradiol: Menopause beyond “Old Wives’ Tales* (Nelson), 17-Beta-Estradiol production depends on complex interactions involving the hypothalamic-pituitary-ovarian axis supporting the growth and development of ovarian follicles. (**Figure 2**) Recent studies have found that 17-Beta-Estradiol can rapidly activate cell signaling *via* membrane receptors and is involved in brain processes.

Preclinical studies in rodents have shown that estradiol can improve working and reference memory and decrease anxiety-like behaviors. Women receiving estradiol exhibit changes in frontal activation during working memory tasks. Adolescent girls and young women deficient in circulating estradiol need estradiol replacement therapy to develop and maintain secondary sex characteristics and bone mass. Women deficient in estradiol are at risk of significant morbidity and mortality related to osteoporosis, cardiovascular disease, and dementia, and even a shortened life expectancy. The best evidence supports using a physiologic approach to replacing 17-Beta-Estradiol by transdermal or transvaginal administration to avoid the “first pass hepatic effects” of oral estrogen administration.

In *Abnormal Trabecular Bone Score, Lower Bone Mineral Density and Lean Mass in Young Women With Premature Ovarian Insufficiency (POI) Are Prevented by Oestrogen Replacement,* Samad et al. found deficits in BMD, trabecular microarchitecture, and lean mass in women with POI. However, hormone replacement protects against declines in these variables. Their evidence supports assessing skeletal and muscle health in POI and the importance of hormonal replacement.

Huang et al. studied *Lipid Profile in Patients With Primary Ovarian Insufficiency: A Systematic Review and Meta-Analysis* and demonstrated that total cholesterol, triglycerides, and LDL levels were significantly higher in women with POI than controls. The authors recommend early medical intervention to minimize the risk of CVD morbidity and mortality associated with dyslipidemia in women with POI. Qiu et al. in *Comparison of the reproductive outcome between 2 and 4 mg daily doses of estradiol after hysteroscopic adhesiolysis: a propensity score matching analysis-retrospective cohort study* found no significant difference in outcome between the two groups. They suggest using the lower dose (2 mg) to minimize side effects.

## What about progesterone?

In *Adaptive, reversible, hypothalamic reproductive suppression: More than functional hypothalamic amenorrhea*, Prior suggests that current concepts of menstrual and ovulation suppressions in women need to be redefined as a continuum that may in some cases be protective, adaptive, and potentially reversible. The work indicates that treatments for these conditions must be updated to fit these new concepts. The article suggests further research on using cyclic progesterone as a therapy and recommends prospective, randomized, controlled trials to investigate this avenue. Overall, this new understanding of women’s reproductive disturbances may provide a positive and more science-based approach to women’s hormonal health (12).

## A final note

Findings in women’s health research, whether supported by public, private, or hybrid funding mechanisms, continue to be translated to the clinical arena. This Research Topic includes several examples where endocrinological interventions are supported as strategies to improve women’s health and well-being. Specifically with regards to POI and the loss of endogenous ovarian hormone production, we note that more and more clinically actionable information continues to be produced about how HRT can be used to alleviate symptoms and improve health. We must press for increased public funds for women’s health research to correct obvious and impactful health disparities between women and men, and creative partnerships between public and private entities are an additional way we can continue to narrow the gap.

## Author contributions

LN: Writing – review & editing, Writing – original draft, Visualization, Resources, Project administration, Investigation, Formal Analysis, Conceptualization. JJ: Writing – review & editing.
